# Interictal and postictal ^18^F-FDG PET/CT in epileptogenic zone localization

**DOI:** 10.1590/0100-3984.2021.0141

**Published:** 2022

**Authors:** Marcela Santos Carvalho, Marina Koutsodontis Machado Alvim, Elba Etchebehere, Allan de Oliveira Santos, Celso Dario Ramos, Juliana Luz Passos Argenton, Fernando Cendes, Bárbara Juarez Amorim

**Affiliations:** 1 Medicina Nuclear Diagnóstico (MND), Campinas, SP, Brazil.; 2 Departamento de Neurologia, Faculdade de Ciências Médicas da Universidade Estadual de Campinas (FCM-Unicamp), Campinas, SP, Brazil.; 3 Serviço de Medicina Nuclear, Departamento de Anestesiologia, Oncologia e Radiologia (DAOR), Faculdade de Ciências Médicas da Universidade Estadual de Campinas (FCM-Unicamp), Campinas, SP, Brazil.; 4 Departamento de Bioestatística, Universidade Estadual de Campinas (Unicamp), Campinas, SP, Brazil.

**Keywords:** Positron emission tomography computed tomography/trends, Epilepsy/diagnostic imaging, Fluorodeoxyglucose F18/administration & dosage, Electroencephalography/methods, Magnetic resonance imaging, Tomografia por emissão de pósitrons combinada à tomografia computadorizada/tendências, Epilepsia/diagnóstico por imagem, Fluordesoxiglucose F18/administração & dosagem, Eletroencefalografia/métodos, Ressonância magnética

## Abstract

**Objective:**

To evaluate the performance of ^18^F-fluorodeoxyglucose positron-emission tomography/computed tomography ( ^18^F-FDG PET/CT) in localizing epileptogenic zones, comparing ^18^F-FDG injection performed in the traditional interictal period with that performed near the time of a seizure.

**Materials and Methods:**

We evaluated patients with refractory epilepsy who underwent ^18^F-FDG PET/CT. The reference standards for localization of the epileptogenic zone were histopathology and follow-up examinations (in patients who underwent surgery) or serial electroencephalography (EEG) recordings, long-term video EEG, and magnetic resonance imaging (in patients who did not). The ^18^F-FDG injection was performed whether the patient had an epileptic seizure during the EEG monitoring period or not. The ^18^F-FDG PET/CT results were categorized as concordant or discordant with the reference standards.

**Results:**

Of the 110 patients evaluated, 10 were in a postictal group (FDG injection after a seizure) and 100 were in the interictal group. The ^18^F-FDG PET/CT was concordant with the reference standards in nine (90%) of the postictal group patients and in 60 (60%) of the interictal group patients. Among the nine postictal group patients in whom the results were concordant, the ^18^F-FDG PET/CT showed hypermetabolism and hypometabolism in the epileptogenic zone in four (44.4%) and five (55.6%), respectively.

**Conclusion:**

Our data indicate that ^18^F-FDG PET/CT is a helpful tool for localization of the epileptogenic zone and that EEG monitoring is an important means of correlating the findings. In addition, postictal ^18^F-FDG PET/CT is able to identify the epileptogenic zone by showing either hypometabolism or hypermetabolism.

## Abstract (pt)

**Objetivo:**

Avaliar a capacidade da PET/CT FDG detectar a zona epileptogênica, com injeção da FDG realizada tanto no período interictal como perto de uma crise epiléptica.

**Materiais e Métodos:**

Foram avaliados pacientes com epilepsia de difícil controle que realizaram PET/CT FDG. A zona epileptogênica foi definida pelo *follow up*/anatomopatológico ou eletroencefalogramas (EEGs) seriados, telemetria e ressonância magnética. PET/CT FDG foi realizada independentemente se o paciente tinha crise epiléptica durante a monitoração com EEG ou no período interictal. Os resultados foram definidos como concordantes ou discordantes, comparando com a zona epileptogênica.

**Resultados:**

Foram incluídos no estudo 110 pacientes: 10 no grupo pós-ictal (injeção de FDG depois da crise) e 100 no grupo interictal. A PET/CT FDG foi concordante com a zona epileptogênica em nove pacientes do grupo pós-ictal (90%) e 60 pacientes do grupo interictal (60%). Entre os nove pacientes concordantes do grupo pós-ictal, quatro mostraram hipermetabolismo (44,4%) e cinco mostraram hipometabolismo na zona epileptogênica (55,6%).

**Conclusão:**

Nossos resultados confirmaram que a PET/CT FDG é uma ferramenta útil na localização da zona epileptogênica e a monitoração com EEG é muito importante para correlacionar os achados. Além disso, PET/CT FDG realizada no período pós-ictal é capaz de identificar a zona epileptogênica, mostrando tanto hipometabolismo como hipermetabolismo.

## INTRODUCTION

Epilepsy affects more than 70 million people worldwide, and the estimated proportion of the general population with active epilepsy (i.e., with recurrent seizures or requiring continual treatment) at any given time is 0.4-1.0%. However, some studies have suggested that the proportion is higher (0.7-1.5%) in low- and middle-income countries^(1)^. In cases of epilepsy that are refractory to treatment (even optimized pharmacological treatment), surgery is warranted and localization of the epileptogenic zone (EZ) through neuroimaging examinations is imperative.

One well-established imaging tool for EZ localization is ^18^F-fluorodeoxyglucose positron emission tomography/computed tomography ( ^18^F-FDG PET/CT), which is employed to facilitate the surgical decision-making process in more than 30% of cases^(2,3)^. The use of ^18^F-FDG PET/CT provides information that anatomical methods such as magnetic resonance imaging (MRI) can miss, and the area of hypometabolism identified on an ^18^F-FDG PET/CT scan may be larger than the area of the anatomical lesion identified on MRI^(4)^. It is even possible that ^18^F-FDG PET/CT with detect an area of hypometabolism consistent with an EZ where MRI has shown a typical brain structure^(5,6)^. A seizure-free outcome after surgery is less likely in patients without anatomical lesions. However, when ^18^F-FDG PET/CT detects an area of hypometabolism, despite a negative MRI result, the prognosis is similar to that for patients with anatomical lesions identified on MRI (i.e., good). In patients who present with multiple structural lesions, ^18^F-FDG PET/CT can help define which of the lesions is responsible for the seizures^(7,8)^.

An ^18^F-FDG PET/CT examination performed during the interictal period is used in order to identify areas of hypometabolism^(9)^. However, injecting the radiotracer near the time of a seizure can reveal an area of hypermetabolism. A finding of hypermetabolism on ^18^F-FDG PET/CT of a patient with epilepsy is rare, with a reported incidence of 2.2-6.6%, and is an essential indicator of the epicenter of the EZ^(10,11)^. There have been only a few studies analyzing the benefit of performing ictal ^18^F-FDG PET/CT, and all of those were retrospective studies^(12-15)^.

In this study, we aimed to investigate, prospectively, the performance of ^18^F-FDG PET/CT in localizing the EZ in patients with refractory epilepsy who are candidates for surgery, comparing ^18^F-FDG injection performed in the traditional interictal period with that performed near the time of a seizure.

## MATERIALS AND METHODS

### Patients

In this prospective study, we enrolled patients with epilepsy that was refractory to clinical treatment between January 2015 and July 2017, all of the patients underwent ^18^F-FDG PET/CT as part of the investigation to localize the EZ. Patients in whom there were any technical problems in imaging acquisition were excluded, as were those in whom the data were insufficient to define the EZ.

All procedures were performed in accordance with the policies of the local human subject protection committee. The local institutional review board approved the study (Reference no. 02607618.3.0000.5404), and all participating patients gave written informed consent.

### Reference standards for localization of the EZ

The criteria employed to determine the location of the EZ and define the ^18^F-FDG PET/CT findings, in patients who did and did not undergo surgery, were as follows:

• In patients who underwent surgery, the location of the EZ was determined by histopathological analysis and follow-up examinations.

• In patients not submitted to surgery, a multidisciplinary team of epilepsy experts determined the location of the EZ by consensus. Collectively, the team had over 20 years of experience and was composed of epileptologists, neuroradiologists, neurosurgeons, nuclear medicine physicians, and neuropsychologists. All of the cases were discussed on the basis of the clinical/neurological history, seizure semiology (patient and family description, together with any available videos), serial EEG recordings, long-term video EEG monitoring results, and MRI findings. In addition, functional (language and motor) MRI and neuropsychological tests were performed regarding the localization of the EZ. Some patients also underwent ictal and interictal single-photon emission CT (SPECT) of the brain.

### ^18^F-FDG PET/CT

#### Patient preparation and image acquisition

After being fitted with a indwelling venous catheter, patients remained at rest for 90 min in an EEG room with dim lighting, after which they received an injection of 185 mBq (5 mCi) of ^18^F-FDG. All ^18^F-FDG injections were performed under EEG monitoring. The EEG monitoring started 60 min before ^18^F-FDG injection and ended 30 min after. At 60 min after the ^18^F-FDG injection, patients underwent PET/CT in a dedicated scanner (Biograph 40 TruePoint mCT; Siemens Medical Solutions, Knoxville, TN, USA). The acquisition was performed with one bed position centered over the skull for 5 min, and CT images were used for attenuation correction and anatomical correlation.

The ^18^F-FDG injection was performed whether the patient had an epileptic seizure during the EEG monitoring period or not. Therefore, we separated the patients into two groups: those who were injected with ^18^F-FDG near the time of a seizure during the EEG monitoring period (from 60 min before until 30 min after FDG injection); and those who were injected with ^18^F-FDG but did not present a seizure during that period.

#### Visual and quantitative imaging analysis

All images were interpreted by two nuclear medicine physicians, working independently, and disagreements were resolved by consensus. Areas of hypometabolism and hypermetabolism on ^18^F-FDG PET/CT were considered to be EZs, cerebral hemisphere asymmetries being noted and cortical metabolism being compared with cerebellar metabolism.

The quantitative analysis of ^18^F-FDG PET/CT was performed with Scenium software (Syngo.via Neurology Software Package; Siemens Medical Solutions), which analyzes patient images in comparison with those of age-matched normal individuals in a database. Areas of hypometabolism or hypermetabolism were considered significant if the values were > 2 standard deviations different from those obtained for normal individuals. The ^18^F-FDG PET/CT results regarding the localization of the EZ were categorized in relation to those obtained with the reference standards, and we defined concordance and discordance as follows^(16)^:

- Concordant
  1. Areas of hypometabolism or hypermetabolism overlapping with the EZ localized by the reference standards, even if the area identified by ^18^F-FDG PET/CT extended beyond the predefined area, to other lobes, given that hypometabolism typically extends to areas surrounding the EZ.
  2. Bilateral hypometabolism in mirroring lobes, although with asymmetric occurrence and predominance in the region of the EZ.
- Discordant
  1. Normal ^18^F-FDG PET/CT results.
  2. Areas of hypometabolism or hypermetabolism contralateral to the EZ localized with the reference standards.
  3. Areas of hypometabolism or hypermetabolism in a different lobe altogether, unrelated to the EZ localized with the reference standards.

### Statistical analysis

The Mann-Whitney test was used in order to evaluate the capability of ^18^F-FDG PET/CT to properly localize the EZ, considering the age at onset and frequency of the seizures. Fisher’s exact test was used in order to determine whether there was a significant intergroup difference in terms of the performance of ^18^F-FDG PET/CT in localizing the EZ. The level of significance adopted was 5%. The statistical analysis was performed with the Statistical Analysis System for Windows, version 9.4 (SAS Institute Inc., Cary, NC, USA).

## RESULTS

### Patients

A total of 151 patients with refractory focal epilepsy underwent ^18^F-FDG PET/CT scans. Of those, 41 were excluded because it was not possible to localize the EZ with the reference standards. Therefore, the final sample included 110 patients with epilepsy that was refractory to clinical treatment and who were candidates for surgical treatment. All of the patients had been on polytherapy with different combinations of antiepileptic medications for many years.

The mean duration of clinically intractable epilepsy was 25.3 years (range, 0 to 58 years), the mean age at the onset of seizures was 11.2 years (range, one month to 55 years), and the mean number of seizures per month was 19 (range, 0-600). There were 22 patients who reported having fewer than one epileptic seizure per month; for the statistical analysis, those patients were categorized as having had zero seizures per month. Table 1 shows the demographic and clinical characteristics of the patients. Among the 110 patients evaluated, the EZ was found to be located exclusively in the temporal lobe in 75 (68.2%), exclusively in a lobe other than the temporal lobe in 31 (28.2%), and in more than one lobe (including the temporal lobe) in four (3.6%).

**Table 1:** Characteristics of patients with refractory epilepsy.

Characteristic	(N = 110)
Gender, n (%)	
Male	43 (39.1)
Female	67 (60.9)
Age (years), mean ± SD (range; median)	
At diagnosis	11.2 ± 10.6 (0.1-55; 9)
At inclusion in the study	35.0 ± 15.0 (3-63; 36)
Seizure frequency (n/month), mean (range)	19 (0-600)
Temporal lobe epilepsy, n (%)	64 (58.2)
Extra-temporal lobe epilepsy, n (%)	46 (41.8)

SD, standard deviation.

Of the 110 patients, 24 (21.8%)—three in the group with a seizure during the EEG monitoring period and 21 in the interictal group—underwent surgery. Therefore, the definitive location of the EZ was determined by histopathological analysis and follow-up examinations in those patients. Histopathology revealed hippocampal sclerosis in 10 (41.6%) of those 24 patients, cortical dysplasia in seven (29.2%), gliosis in five (20.8%), oligodendroglial hyperplasia in one (4.2%), and glioma in one (4.2%).

In the 86 patients who did not undergo surgery, the definitive location of the EZ was determined by the multidisciplinary team of epilepsy experts. In all of those patients, the findings were concordant regarding the signs mentioned and the results of the examinations performed (seizure semiology, serial EEG, long-term video EEG monitoring, MRI, functional MRI, neuropsychological tests, and ictal/interictal SPECT).

### ^18^F-FDG PET/CT results

Of the 110 patients in the sample, 10 (9.1%) had a seizure during the EEG monitoring period and 100 (90.9%) did not. The performance of ^18^F-FDG PET/CT in localizing the EZ did not differ significantly between the groups (*p* = 0.85). There was also no significant difference in the ^18^F-FDG PET/CT performance regarding the age at onset (*p* = 0.65) or the frequency of seizures (*p* = 0.75).

#### Postictal group

Among the 10 patients who had a seizure during the EEG monitoring, the reference standard was surgery in three (30%). All 10 patients had only one seizure during the EEG monitoring period, the seizures occurring 3-55 min before ^18^F-FDG injection (postictal PET/CT). None of the patients had an epileptic seizure after injection of the radiotracer. The seizure duration ranged from 10 s to 185 s. Eight patients (80%) had a focal seizure, with impaired awareness and motor manifestation, and two (20%) had an electrographic seizure.

**Figure 1:**
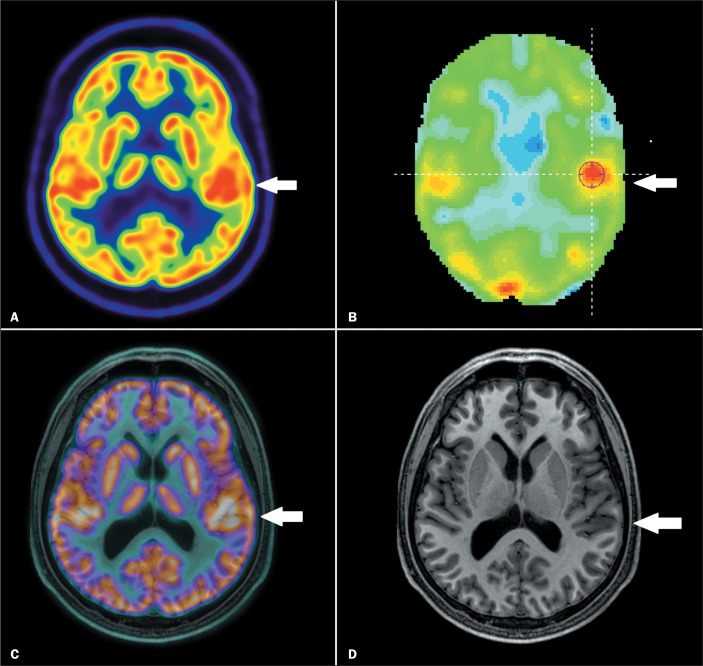
^18^F-FDG PET/CT of a patient in the postictal group, showing hypermetabolism. Axial ^18^F-FDG PET/CT (A), quantitative analysis of the ^18^F-FDG PET/CT (B), and a fused (PET/CT-MRI) image (C), all showing focal hypermetabolism in the left frontotemporal region (arrows). Axial MRI (D) showing an area suspicious for focal cortical dysplasia (arrow), coincident with the area identified on ^18^F-FDG PET/CT. This patient underwent surgery, and the histopathological analysis revealed oligodendroglial hyperplasia.

**Figure 2:**
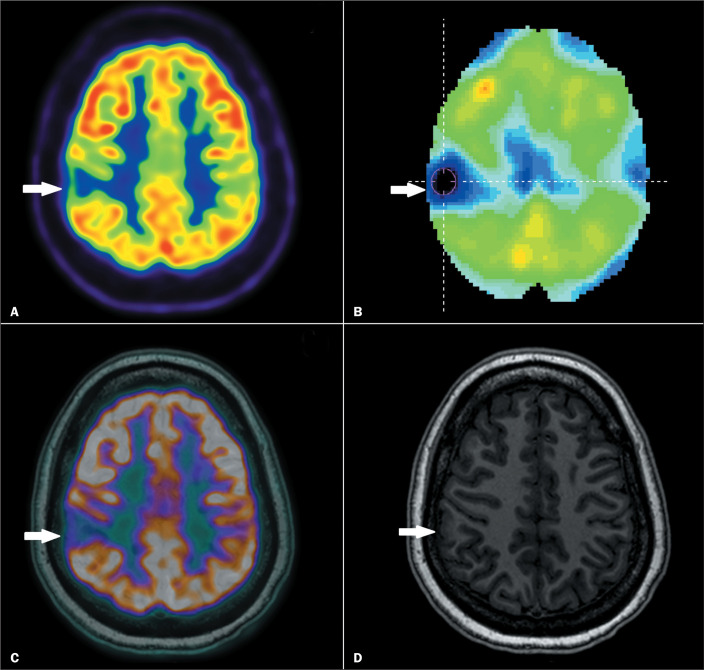
^18^F-FDG PET/CT of a patient in the postictal group, showing hypometabolism. Axial ^18^F-FDG PET/CT (A), quantitative analysis of the ^18^F-FDG PET/CT (B), and a fused (PET/CT-MRI) image (C), all showing focal hypometabolism in the right parietal lobe (arrows). Axial MRI (D) showing an area suspicious for focal cortical dysplasia (arrow), coincident with the area identified on ^18^F-FDG PET/CT. This patient underwent surgery, and the histopathological analysis revealed focal cortical dysplasia.

The ^18^F-FDG PET/CT was concordant with the reference standard in nine (90%) of the patients in the postictal group. Among those nine patients, the ^18^F-FDG PET/CT showed hypermetabolism in the EZ in four (44.4%) and hypometabolism in the EZ in five (55.6%). The ^18^F-FDG PET was concordant with the reference standard in all three of the postictal group patients who underwent surgery: in one, the ^18^F-FDG was injected 42 min after the seizure and the ^18^F-FDG PET/CT showed hypermetabolism in the EZ (Figure 1); in the other two, the ^18^F-FDG was injected 3 and 50 min after the seizure, respectively, and the ^18^F-FDG PET/CT showed hypometabolism in the EZ (Figure 2). The ^18^F-FDG PET/CT was discordant with the reference standard in only one (10%) of the postictal group patients.

In the postictal group as a whole, the mean time from seizure to radiotracer injection was 29.7 min (range, 3-55 min). That mean time was 35.7 min among the four patients in whom the ^18^F-FDG PET/CT showed hypermetabolism in the EZ and 24.2 min among the six patients in whom the ^18^F-FDG PET/CT showed hypometabolism in the EZ.

#### Interictal group

Among the 100 patients in the interictal group, the ^18^F-FDG PET/CT was concordant with the reference standard in 60 (60%), showing hypometabolism in the EZ in 59. Notably, the ^18^F-FDG PET/CT showed hypermetabolism in the EZ in one patient. That patient presented bilateral hypermetabolism, identified visually and quantitatively, in the occipital region white matter, MRI showed bilateral gray matter heterotopia in the posterior periventricular region, and EEG showed bilateral occipital activity.

The ^18^F-FDG PET/CT was discordant with the reference standard in 40 (40%) of the interictal group patients. In 21 (52.5%) of those 40 patients, the ^18^F-FDG PET/CT showed no alterations; in the remaining 19 patients (47.5%), it showed hypometabolism in an area that the reference standard had not identified as an EZ.

## DISCUSSION

To our knowledge, this is the first prospective study in which ^18^F-FDG PET/CT was performed near the time of a seizure and during the interictal period. One of the most important and surprising findings of our study was that postictal ^18^F-FDG PET/CT was better able to localize the EZ than was interictal ^18^F-FDG PET/CT. Postictal ^18^F-FDG PET/CT was concordant with the reference standard in 90% of the patients, whereas interictal ^18^F-FDG PET/CT was concordant in only 60%. However, that difference was not significant, because of the small number of patients in the postictal group.

Interictal ^18^F-FDG PET is a well-established functional imaging tool in refractory epilepsy because it can show hypometabolism in the EZ. Although ^18^F-FDG PET has been used for that purpose for more than two decades, the cause of the hypometabolism remains unclear. Possible explanations include anatomical variations, underlying pathologic processes, an effect caused by repeated seizures, and postictal depression of cerebral activity^(13,17)^. However, interictal ^18^F-FDG PET, which is indicated mainly when no structural lesions are identified on MRI, enhances the detection of type I cortical dysplasia that is MRI negative^(18)^. Depending on the resolution of the PET/CT scanner, the sensitivity of interictal ^18^F-FDG PET/CT to detect hypometabolism varies according to the location of the EZ: in temporal lobe epilepsy, it is nearly 80%^(19,20)^, whereas it is only 52% in frontal lobe epilepsy and only approximately 53% overall^(17)^. Because our sample was composed of patients with temporal or extratemporal epilepsy, the sensitivity of interictal ^18^F-FDG PET/CT to localize the EZ was similar to that reported in the literature.

We defined a postictal ^18^F-FDG PET/CT study as one in which the ^18^F-FDG was injected soon after the seizure. In that setting, two patterns of glucose metabolism were identified in the EZ: hypometabolism and hypermetabolism. The reason why postictal injection produces either hypermetabolism or hypometabolism has yet to be elucidated. In our limited postictal sample, the pattern of glucose metabolism did not seem to be related to the time from seizure to ^18^F-FDG injection. It may be that the slow uptake of ^18^F-FDG by the brain tissue produces mixed ictal/postictal scans^(21)^. Increased glucose metabolism and perfusion in the postictal state are probably related to the increased energy output required for the restoration of resting membrane potentials and chemical homeostasis following an epileptic event^(12)^. In patients with repeated seizures during tracer uptake, hypermetabolism is believed to be associated with the site of the ictal focus but can also be present in areas of secondary seizure propagation.

Our findings underscore the importance of continuous scalp EEG monitoring during ^18^F-FDG injection and uptake, to confirm the interictal state of the patient and to assist in the interpretation of ^18^F-FDG uptake when an ictal scan is recorded^(13)^. During the interpretation of the images, it is also important be aware of the fact that the EZ can present hypometabolism or hypermetabolism and to correlate that with the EEG findings.

There have been only a few studies in which ^18^F-FDG PET was performed during the ictal phase or soon after a seizure^(12-15,21)^. In our sample, the postictal ^18^F-FDG PET/CT scans showed focal hypermetabolism in 3.6% of the patients, which is similar to the 2.0-7.0% reported in other studies^(10,11)^. Shur et al.^(11)^ retrospectively evaluated 317 ^18^F-FDG PET scans and identified hypermetabolism in seven, all of whom presented type I focal cortical dysplasia on histopathology and excellent post-treatment seizure reduction. In a study of 498 ^18^F-FDG PET scans, Bansal et al.^(10)^ identified focal hypermetabolism in 33 patients, 17 of whom underwent surgical resection, after which the histopathological analysis showed malformation. Chugani et al.^(12)^ studied seven children in whom ^18^F-FDG PET showed hypermetabolism in the EZ, and three of those children had seizures ≤ 15 min before ^18^F-FDG injection (therefore undergoing postictal ^18^F-FDG PET). The other four patients underwent interictal ^18^F-FDG PET, although the authors reported that EEG revealed spike-and-wave activity in those patients. The regions of hypermetabolism corresponded to the suspected EZs determined by EEG. In another study, Chugani et al.^(14)^ examined 18 children who had a seizure immediately after ^18^F-FDG injection, during the uptake period, and who presented hypermetabolism on ^18^F-FDG PET images; among these, seven patients with lateralization showed a relatively small area of hypermetabolism that was coincident with the EZ localized by ictal EEG.

In the present study, most (55%) of the postictal ^18^F-FDG PET/CT examinations showed hypometabolism in the EZ and were concordant with the reference standard. Hypometabolism most likely represents postictal depression of cerebral activity^(13)^. Because there was only one discordant result in the postictal group, we were unable to compare the concordant and discordant examinations in terms of the time from seizure to ^18^F-FDG injection. Barrington et al.^(13)^ also observed hypometabolism in most (four of six) ^18^F-FDG PET/CT studies performed near the time of a seizure, although their patients had seizures after ^18^F-FDG injection.

Our study has some limitations. First, despite the relatively large patient sample, only a small number of patients underwent postictal ^18^F-FDG PET/CT. Because this was a prospective study, the incidence of ictal events during the EEG monitoring period was low. Second, only 21.8% of patients underwent surgery for EZ resection. There is a need for further studies involving larger numbers of patients undergoing postictal ^18^F-FDG PET/CT, as well as performing interictal ^18^F-FDG PET/CT in patients who have undergone postictal ^18^F-FDG PET/CT, which will allow the postictal and interictal EZ metabolism to be compared.

Our data confirm that ^18^F-FDG PET/CT is a helpful tool in EZ localization and that EEG monitoring should be performed in order to compare the ^18^F-FDG PET/CT findings with the EEG findings. We speculate that postictal ^18^F-FDG PET/CT could be an alternative for localizing the EZ in clinical practice. When a seizure occurs during an ^18^F-FDG PET/CT performed in a patient with epilepsy, some physicians cancel the acquisition or the subsequent analysis of the images. The present study shows that one should not discard or discount such images, which could perform well in the localization of the EZ. A postictal ^18^F-FDG PET/CT is able to identify the EZ, showing either hypometabolism or hypermetabolism. In that setting, it is quite important to perform EEG monitoring in order to compare the ^18^F-FDG PET/CT findings with the EEG findings. Further studies with larger patient samples are needed in order to confirm our findings.
